# Scientific Items

**Published:** 1877-03-20

**Authors:** 


### SCIENTIFIC ITEMS.

*The Wonders of a Hen's Egg.*—In twelve hours of setting, points indicating life in the egg appear. On the second day the heart may be seen to beat having a horse-shoe shape, but as yet no red blood. At the fiftieth hour, one auricle of the heart appears. The beating of the heart is first seen in the auricle, and afterwards in the ventricle. At the end of seventy hours, two bubbles are seen for the brain, one for the bill and two for the fore and hind parts of the head. The liver may be detected on the 5th day. On the 7th day the ventricles and circulation are complete and the brain has consistency, from which time the various organs are rapidly developed and the chicken mature at the 18th day.

*Nitro-Glycerine* was discovered in 1848 by Sobrers, a pupil of Pelouze, but it was not used until 1864 that Alfred Nobel obtained a patent for its use in blasting.

*Dynamite*, consists of Nitro-Glycerine and a kind of selicious or infusorial earth known under the names of selicious marl, tripoli, rotten-stone, etc. This earth absorbs the nitro-glycerine without destroying it, and the result is a mixture which is not liquid, and can be transported with much less risk.

*Giant Powder* is dynamite adulterated with nitrate of soda or potash. A preparation called *rend-rock* is a mixture of gun powder and dynamite.

*Seed* 1600 *years old.*—In the mines of Laurium, in Greece, which were worked about 1600 years ago, a quantity of seed were discovered, which, when planted under conditions favorable for germination, grew and proved to be a lost species of the genus glancium, (horned poppy.) Pliny and Dioscorides, frequently allude to this beautiful plant in their writings.

*Salt Water made fresh.*—Capt. Edmunds, an Englishman, has patented a machine for obtaining fresh water from sea water. It first converts it into steam which is passed into a condensing chamber, and thence in the form of fresh water to a tank, whence it is drawn off by a tap as required.

*Deaf and Dumb.*—Out of the sixty-seven inmates of the Illinois Deaf and Dumb Asylum, forty-eight are children of first cousins.

